# Isocitrate Dehydrogenase 1 Mutation and Ivosidenib in Patients With Acute Myeloid Leukemia: A Comprehensive Review

**DOI:** 10.7759/cureus.44802

**Published:** 2023-09-06

**Authors:** Adarsh Vardhan Tangella, Ashwin Gajre, Vivek Varma Kantheti

**Affiliations:** 1 Internal Medicine, Andhra Medical College and King George Hospital, Visakhapatnam, IND; 2 Internal Medicine, Lokmanya Tilak Municipal Medical College, Mumbai, IND; 3 Internal Medicine, Rangaraya Medical College, Kakinada, IND

**Keywords:** oncology, idh-mutated, leukemia, aml, ivosidenib

## Abstract

Acute myeloid leukemia (AML) arises from immature myeloid progenitors, resulting in a stem-cell-like proliferative state. This leads to excessive pools of immature cells that cannot function, which usually happens at the cost of the production of mature functional cells, leading to deleterious consequences. The management of AML has intensified as newer targeted therapies have come into existence owing to deeper genetic analysis of the disease and patients. Isocitrate dehydrogenase (IDH) is a cytosolic enzyme that is a part of the Krebs cycle and is extremely important in maintaining the homeostasis of the cell. It is produced by two different genes: IDH1 and IDH2. Ivosidenib has been associated with IDH1 inhibition and has been studied in numerous cancers. This review highlights the studies that have dealt with ivosidenib, an IDH1 inhibitor, in AML, the side effect profile, and the possible future course of the drug. After a scoping review of the available literature, we have identified that studies have consistently shown positive outcomes and that ivosidenib is a promising avenue for the management of AML. But it also has to be kept in mind that resistance to IDH inhibitors is on the rise, and the need to identify ways to circumvent this is to be addressed.

## Introduction and background

Isocitrate dehydrogenase: role, mutations, and pathophysiology

Isocitrate dehydrogenase (IDH) is a cytosolic enzyme responsible for converting isocitrate to alpha-ketoglutarate in the Krebs cycle [1]. This step involves the reduction of nicotinamide adenine dinucleotide phosphate (NADP) to nicotinamide adenine dinucleotide phosphate hydrogen (NADPH) [1]. This step has a very crucial role in maintaining the free radical levels and the formation of reactive oxygen species in the cell, which help in providing anti-bacterial abilities to the cell [2]. Alpha-ketoglutarate is also an anaplerotic substrate that contributes to mitochondrial adenosine triphosphate (ATP) synthesis, maintaining the cell's energy balance [1]. Wild-type IDH (wtIDH) is usually found in all living organisms and is responsible for the above-mentioned process. It is usually synthesized as a result of the activation of two genes: IDH1 and IDH2 [3,4]. Studies have shown that the IDH 1 mutation is more frequently seen in acute myeloid leukemia (AML) than the IDH 2 mutation [5].

IDH1 mutations (mtIDH1) are seen in 10-20% of AML patients [6,7]. Although some studies talk about poorer prognostic rates in mtIDH1 AML patients [8], there is evidence that this status does not affect outcomes [9], making the discussion equivocal. Apart from AML, IDH1 mutations are also seen in more than 70% of glioblastomas [6], more than 55% of chondrosarcomas [10], about 13% of intrahepatic cholangiocarcinomas [11], and about 10% of malignant melanomas [12]. Ivosidenib is a drug that selectively inhibits mtIDH1 and has been extensively studied in a wide range of malignancies [5-7]. Trials using ivosidenib have been done in most of these malignancies and have shown improved outcomes, especially in cholangiocarcinoma [13] and glioblastoma [14], and their outcomes have been described in Table 1 later. Although numerous small molecule inhibitors have been tested preclinically for inhibition of IDH1, AG 120 - now referred to as ivosidenib - was the only drug that was granted FDA approval.

**Table 1:** Ivosidenib in cholangiocarcinoma and glioblastoma

Malignancy	Median progression-free survival (PFS) in ivosidenib arm	Additional study outcomes
Cholangiocarcinoma [13]	2.7 months (vs. 1.4 months in the placebo arm)	Median follow up for PFS - 6.9 months, No treatment-related deaths.
Glioblastoma [14]	Non-enhancing gliomas - 13.6 months enhancing gliomas - 1.4 months	Stable disease in 85.7% patients with non-enhancing glioma and 45.2% patients with enhancing glioma

**Figure 1:**
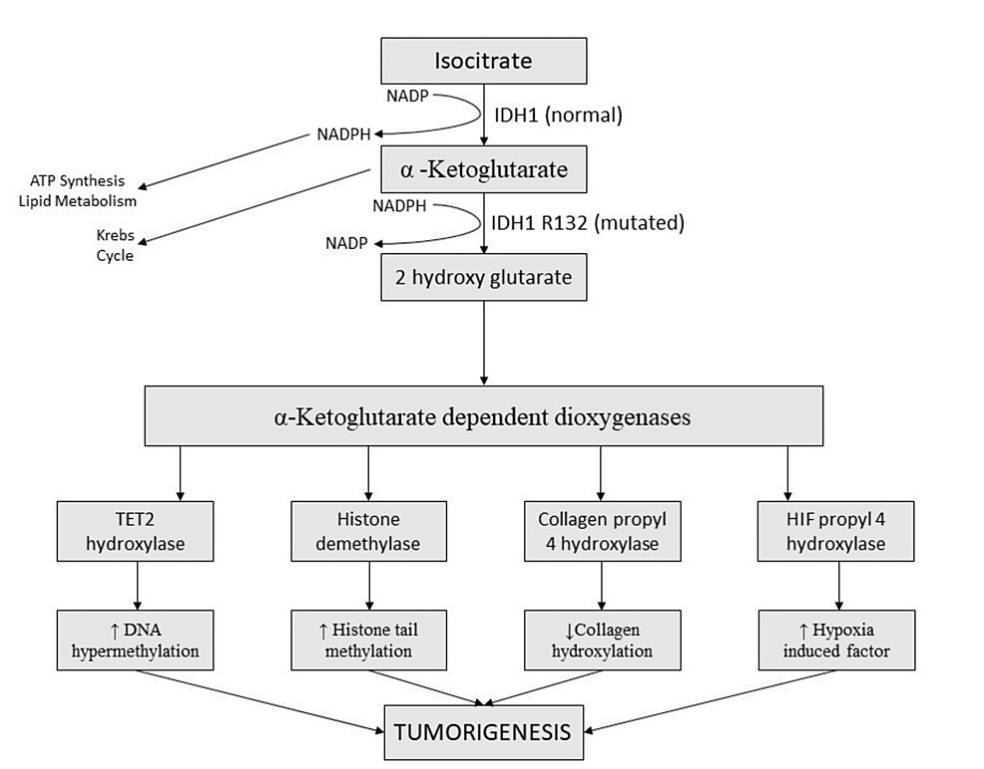
Flowchart showing the mechanism of tumorigenesis in isocitrate dehydrogenase 1 mutation NADP: nicotinamide adenine dinucleotide phosphate, NADPH: nicotinamide adenine dinucleotide phosphate hydrogen, IDH1: isocitrate dehydrogenase isoform type 1, ATP: adenosine triphosphate, TET2: tet methylcytosine dioxygenase 2, HIF: hypoxia-induced factor

The classic mutation of IDH1 occurs at arginine 132 and is referred to as IDH1 R132 [15]. This mutated form of IDH1 leads to aberrant conversion of alpha-ketoglutarate into D-2-hydroxyglutarate (2-HG), which is an oncometabolite in this pathway [15]. This conversion also utilizes NADPH, leading to its conversion into NADP, which depletes the NADPH levels in the cell, causing inadequate generation of reactive oxygen species and also insufficient lipid metabolism, ultimately disturbing the internal milieu of the cell [15,16]. 2-HG has an inhibitory effect on certain enzymes such as tet methylcytosine dioxygenase 2 (TET2) hydroxylase, histone demethylase, collagen propyl 4 hydroxylase, and HIF propyl 4 hydroxylase. Inhibition of TET2 hydroxylase leads to increased DNA hypermethylation. Inhibition of histone demethylase leads to increased histone tail methylation. Collagen propyl-4 and HIF propyl-4 hydroxylase inhibition leads to elevated levels of hypoxia-inducing factor (HIF) and reduced collagen hydroxylation. All these enzymatic errors lead to global hypermethylation without gene expression, causing the arrest of cellular maturation [15-17]. Hypermethylation causes inhibition of promoters responsible for the activation of tumor suppressor genes, leading to uncontrolled cell proliferation [16,17]. The following Figure 1 shows the connection between the IDH1 mutation and tumorigenesis.

Ivosidenib: pharmacokinetics, pharmacodynamics, and scope of usage in cancer

Ivosidenib has been extensively studied before being used in clinical trials to determine the best route of administration, its effects on the P450 enzyme complex, and ways to improve efficacy. A single 10 mg/kg dose of ivosidenib orally showed good absorption and bioavailability of 48% in preclinical studies and was hence used as a convenient oral drug, improving patient compliance [18]. Although it did have good bioavailability, it has poor central nervous system (CNS) penetration and hence might probably be ineffective if given orally to CNS tumors. It is primarily metabolized in the liver. Four major metabolites were identified, mostly hydroxylated forms, but the biological activity of these forms has yet to be studied. Ivosidenib is associated with weak direct inhibition of CYP2C8, CYP2C19, CYP2D6, and CYP3A4/6. It also causes an increase in CYP2B6 activity [18].

Ivosidenib has been approved by the FDA for use in unresectable or refractory cholangiocarcinoma [19], relapsed or refractory acute myeloid leukemia [20], and glioblastoma [14]. Table 1 summarizes the outcomes of studies pertaining to ivosidenib in cholangiocarcinoma [13] and glioblastoma [14]. Studies related to acute myeloid leukemia will be the scope of this review and will be subsequently discussed in detail.

## Review

Clinical trials and studies in acute myeloid leukemia using ivosidenib

Ivosidenib Monotherapy in Newly Diagnosed IDH1 Mutant AML

A study by Roboz et al. analyzed how patients who are ineligible for the usual intensive regimen but have a targetable IDH1 mutation can become ideal candidates for ivosidenib monotherapy. Frailty due to age was the biggest factor that prevented these patients from receiving an intensive 7+3 chemotherapy regimen. All of them had IDH1 mutations. This was a subset analysis of a larger phase 1 study involving IDH1 mutant hematological malignancies in which 34 patients out of 258 were not eligible for standard therapy. They received 500 mg of ivosidenib every day for 4.5 months. An important observation in this group was that 26 patients had secondary AML, and 16 received more than or equal to one hypomethylating agent for an antecedent hematologic disorder. On analyzing the responses, the complete remission (CR) plus complete remission with partial hematological response (CRh) rates were 42.4%, out of which the CR was 30.3%. About 62% of total CR+CRh patients and 78% of CR patients remained in remission at the end of one year. About 9/21 transfusion-dependent patients became independent. A very crucial aspect of this study was the IDH1 mutation clearance rates. mtIDH1 clearance was seen in 9/14 patients who were CR+CRh, of whom 5/10 had CR and 4/4 had CRh. This supports the idea that ivosidenib monotherapy can alter the biological state of IDH1, hence leading to a prolonged durable response, especially in patients who might not be eligible for standard therapy due to frailty [21].

Ivosidenib Monotherapy in Relapsed or Refractory IDH1 Mutant AML

A phase 1 study by DiNardo et al. has shown the beneficial effects of using ivosidenib monotherapy specifically in relapsed or refractory AML. Although it was primarily a study meant to understand the efficacy and safety profile of ivosidenib, response evaluation was done, especially in the primary efficacy population of the study. Out of 125 patients, the rate of CR or CRh was 30.4%, and the rate of CR was 21.6%. The overall response rate was 41.6%. The median duration of CR or CRh was 8.2 months, and the median duration of CR was 9.3 months. Out of 84 transfusion-dependent patients in the primary efficacy group, 29 became transfusion-independent for 56 days or more during treatment. This also supports the idea that ivosidenib might have a biological alteration effect on mtIDH1, resulting in a durable, long-term response [22].

*Ivosidenib in Combination With Azacitidine in IDH1 Mutate* *d AML*

In a multicenter phase 3 randomized control trial involving 146 patients randomized to ivosidenib + azacitidine (72) versus placebo + azacitidine arm (74), at the median follow-up period of 12.4 months, the probability of remaining event-free at 12 months was 37% in the ivosidenib arm versus 12% in the placebo arm, making the event-free survival significantly longer in the ivosidenib arm. The median overall survival (OS) was significantly longer with ivosidenib + azacitidine (24 months) versus 7.9 months with placebo + azacitidine. The ivosidenib arm saw a higher rate of differentiation syndrome, bleeding events, and neutropenia, whereas the placebo arm saw a higher incidence of febrile neutropenia and infections. This showed that combining ivosidenib with azacitidine in IDH1 mutant AML patients will ensure a better, longer overall survival, and based on previously discussed phase 1 trials, it might also be associated with a prolonged sustained response [23].

Ivosidenib in Combination With Intensive Chemotherapy in Newly Diagnosed AML

In a multicentre phase 1 study, the addition of ivosidenib or enasidenib (mtIDH2 inhibitors) to a time-tested intensive chemotherapy regimen of 7+3 (cytarabine and an anthracycline) showed that patients tolerated these drugs well without a high incidence of severe adverse effects. The incidence of IDH-induced differentiation syndrome was low due to the addition of cytotoxic chemotherapy. The end-of-induction CR rates for ivosidenib were 55%, and CR with incomplete neutrophil or platelet recovery were 72% and 63%, respectively. In patients with the best response, about 39% of them had IDH1 mutation clearance (which is in line with the deep durable response after ivosidenib mentioned earlier), and 80% of them turned negative for measurable residual disease by flow cytometry [24].

Ivosidenib as a Bridge to Allogeneic Stem Cell Transplant in Refractory and Relapsed AML

Allogeneic stem cell transplant (allo-SCT) has been the mainstay therapy for patients with relapsed or refractory AML. The outcomes of the addition of ivosidenib as an additional therapy alongside allo-SCT have never been analysed before. A retrospective analysis was done by Genthon et al., where they identified that IDH inhibitor therapy can be helpful in preventing the progression of the disease and also in achieving a deeper, durable molecular response post-stem cell transplantation. After a median follow-up since diagnosis of 24.7 months, 9 out of 11 patients who got IDH inhibitors and allo-SCT were alive. The two- and three-year overall survival rates were 100% and 85.7%, respectively, and mtIDH was not detectable in the NGS of all the patients who received allo-SCT [25].

Ivosidenib resistance: mechanism and ways to circumvent it

**Figure 2:**
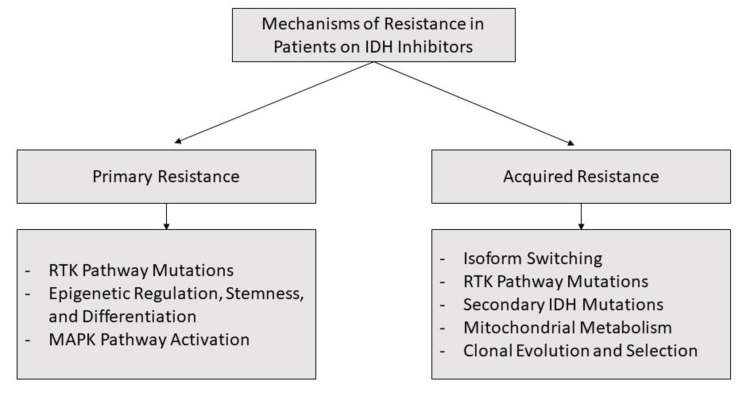
Mechanisms of resistance in patients on IDH inhibitors RTK: receptor tyrosine kinase, MAPK: mitogen-activated protein kinase, IDH: isocitrate dehydrogenase

Ivosidenib resistance is a huge factor responsible for treatment failure and relapses in IDH-mutated cancers [26]. Figure 2 grossly classifies the mechanisms of IDH resistance.

Receptor Tyrosine Kinase (RTK) Pathway Mutations

In a study performed by Choe et al., they observed that around 20 patients out of 74 patients with relapsed or refractory AML with an IDH1 mutation also had RTK pathway mutations [27]. A mutation in one gene, such as neuroblastoma RAS, or in the whole RTK pathway gene group (NRAS, KRAS, PTPN11, KIT, and FLT3) resulted in treatment failure or poor treatment outcomes. This is because, despite being able to block the IDH1-related tumorigenesis pathway, the existence of an additional proliferative pathway probably made the cells depend on it for further proliferation, leading to treatment resistance. This has been proven by studies that have shown that combining FLT3 and IDH inhibitors can increase the complete remission rate from 33% to 42% [28].

Mitogen-Activated Protein Kinase (MAPK) Signaling Pathway Activation

MAPK signal transduction cascade primarily acts through the Ras/Raf/MEK/ERK pathway [29], and activating mutations in MAPK pathway-associated genes such as NRAS, PTPN11, SRSF2, and ASXL1 [30] might result in IDH inhibitor resistance, primarily to IDH2 inhibitors more than IDH1. Molecular cross-interactions between the MAPK pathway and other oncogenic cascades also contribute to tumour progression and drug resistance [31]. Simultaneous mutations in any of the above-mentioned tumorigenesis pathways alongside IDH2 mutations might result in failure of treatment when IDH inhibitors are used due to the presence of an alternate proliferative cascade [27,29]. MEK forms a crucial part of the Ras/Raf tumorigenesis process, which can be targeted by inhibitors such as trametinib along with IDH inhibitors, which might relieve the drug resistance partly or completely [32,33]. It must be noted that in some cases, a mutation in the RAS pathway might not cause complete drug resistance, and some patients might still achieve complete remission despite having RAS mutations alongside IDH mutations. This phenomenon has been studied in NRAS mutations [27,33].

Epigenetic Regulation and Role in Leukemic Stemness

We have already mentioned that IDH1 inhibitors cause the differentiation of cells into mature forms. This process is affected due to epigenetic modifications and coexisting genetic alterations. Promoters of transcriptional regulation of stem cells show extensive hypermethylation, which results in an insufficient treatment response in mIDH1/IDH2 AML [34]. Alongside these changes, mutations in differentiation-related transcription factors such as RUNX family transcription factor 1 (RUNX1) and GATA binding protein 2 (GATA2) are associated with treatment resistance [35,36]. The main mechanism by which these aberrations lead to inadequate responses is a consequence of the inability of the cell to undergo differentiation, which is primarily due to the stem-cell-like proliferative nature (stemness) induced as a result of the widespread epigenetic changes in the genome [37,38]. It is said that about 25% of the patients (15 out of 60) suffer from resistance due to leukemic stemness [34]. Apart from the above-mentioned mechanisms, 2-HG can cause impaired myeloid differentiation and enhanced immature cell surface marker expression, which is a consequence of the hypermethylation of signals responsible for the inhibition of the WNT pathway [39]. Fortunately, epigenetic alterations can be dealt with using therapeutic interventions [23]. Azacytidine specifically targets DNA hypermethylation by inhibiting DNA methyl transferase, and as discussed earlier, by combining mIDH1/2 inhibitors with azacytidine, the OS and PFS in patients with mIDH1/2 AML can be increased and resistance can be circumvented at least to a certain extent [23].

Secondary IDH Mutations

In a study performed by Gatto et al., it was observed that after a prolonged sustained response to IDH1/2 inhibitors, there was a sudden spike in 2-HG levels in a few patients [40,41]. When this was investigated and genomic analysis was performed, it was observed that there were point mutations observed at the drug binding sites of enasidenib and/or ivosidenib that resulted in drug resistance [41]. The substitution of glutamine with glutamate at the binding site of enasidenib (Q316E) caused reduced hydrogen bond formation with the drugs, and the change of isoleucine to methionine (I319M) in IDH2 mutants or serine to phenylalanine (S280F) in IDH1 mutants led to more bulky amino acid residues at the binding site, making it difficult for IDH1/2 inhibitors to bind due to steric hindrance [41]. The only way to go around these mutations is to use novel IDH1/2 inhibitors that do not use the traditional binding sites for their actions. IDH305 [42] or olutasidenib [43] may be helpful in patients with the S280F mutation. LY3410738 is a drug under investigation that can bind to a completely different site on IDH1 (C269) and has exhibited more potent inhibitory activity when compared to ivosidenib [44]. Another mIDH2 inhibitor under investigation is SH1573, which has a lower probability of developing resistance as it does not bind to key residues such as Q316 and I319 [45].

Isoform Switching

IDH has three isoforms, out of which IDH1, or cytosolic IDH, and IDH2, or mitochondrial IDH, are commonly mutated in AML [34]. Usually, patients with IDH-mutant AML have either IDH1 or IDH2 mutations, making them targetable [39]. However, a few patients with mIDH1 AML under follow-up who have shown a durable response with ivosidenib suddenly showed elevated 2-HG levels after long-term therapy [46]. When the genomic analysis was performed, it was noted that these patients underwent isoform switching, where the dominant mutant IDH form changed from mIDH1 to mIDH2, resulting in a relapse of disease and a rapid rise in 2-HG levels. This was specifically observed in patients with IDH1 R132C who ended up developing a neomorphic mutation in IDH2 R140Q. Switching was also observed in patients with IDH1 R132C to IDH2 R172V and IDH2 R140Q to IDH1 R312C. These changes result in resistance to the drug that the patient has been receiving [46]. An interesting conclusion from these findings is the co-existence of mIDH isoforms together in the cell, and the driver of the disease is the isoform that is predominant [46]. A large cohort study published in 2020 observed a 12% isoform switching rate from mIDH1 to mIDH2 among 74 patients [27]. This can be tackled by alternating therapy between enasidenib and ivosidenib, but due to the expense and low response rate, this combined therapy is not a practically feasible option [46]. Another way to obtain a rapid response in these patients is by using vorasidenib, which is a dual mIDH1/2 inhibitor [46]. The rising incidence of drug resistance secondary to isoform switching shows that developing more dual IDH1/2 inhibitors is necessary, which might lead to better and longer treatment outcomes.

Clonal Evolution and Selection

Somatic mutations play a crucial role in disease progression and disease outcomes in cancer [47]. The accumulation of these mutations makes the tumour clonally heterogeneous. The presence of multiple heterogeneous mutations that can be targetable or non-targetable results in the disease becoming treatment-resistant and demanding combination therapies [48]. These mutations, as a result, convert a treatment-sensitive cell to a treatment-resistant form [48]. A study performed by Quek et al. in patients with relapsed refractory AML showed increased variant allele frequency in the colony-stimulating factor 3 receptor (CSF3R) gene, FLT3, and Cbl proto-oncogene (CBL), which is a negative regulator of cytokine signaling. Other concurrent mutations observed in these cases are U2 small nuclear RNA auxiliary factor 1 (U2AF1) and hematopoietic transcription factors such as RUNX1, BCL6 corepressor like 1 (BCORL1), and GATA2 [49]. An integrated genomic analysis of 60 IDH1/2-mutated AML patients showed that clonal evolution causing relapse was associated with multiple biological processes such as the RAS signalling pathway, chromatin structure, hematopoietic transcription factors, and DNA methylation pathways [34]. These aberrations are indeed difficult to manage due to the lack of targeted agents as of now, but if they co-exist with other targetable mutations, the intensity of the disease might be reduced by combining traditional chemotherapeutic regimens with novel agents.

Mitochondrial Metabolism

Mitochondrial metabolism forms a crucial factor for the development of treatment resistance as it is the only mechanism that has a non-genetic pathophysiology. In tumours with mutated IDH1/2, it has been observed that the tumour cells dependence on mitochondrial metabolism for energy is enhanced by multiple folds [50-53]. IDH is responsible for multiple physiological processes, which include fatty acid oxidation (FAO), oxidative phosphorylation (oxPhos), lipid biosynthesis, and many more. Studies have noted that ivosidenib has not been able to satisfactorily inhibit two biological processes: beta-oxidation of fatty acids (FAO) and oxPhos [54]. It was also noted that these cells have enhanced rates of oxPhos, which was proven by increased levels of enzymes, which are a part of the process performed by transcriptomic analysis [54]. AML cells also depend strongly on FAO to provide substrates for the increased oxPhos rates. All these findings suggest that it would be ideal to combine IDH1/2 inhibitors with oxPhos inhibitors, or electron transport chain inhibitors (ETCi) [54]. Table 2 summarizes various mechanisms of resistance and the interventions available to overcome them.

**Table 2:** Summary of ways to circumvent various IDH inhibitor resistance mechanisms RTK: receptor tyrosine kinase, FLT: FMS-related tyrosine kinase 3 ligand, IDH: isocitrate dehydrogenase, MAPK: mitogen-associated protein kinase, ETC: electron transport chain

Mechanism of resistance	Interventions to overcome
RTK pathway mutations	Combination of FLT3 and IDH inhibitors [28]
Epigenetic alterations	Combination of azacytidine and IDH inhibitors [23]
MAPK pathway mutations	Combination of MEK and IDH inhibitors combination of STAT5 and IDH inhibitor [55]
Secondary IDH mutations	Usage of IDH inhibitors with a different binding site on the enzyme - Olutasidenib [42-45]
Isoform switching	Alternating IDH inhibitors or Usage of dual IDH1/2 inhibitors [46]
Clonal evolution	Combination of traditional chemotherapeutic regimen with IDH inhibitors [34,49]
Mitochondrial metabolism	Combination of IDH inhibitors with ETC complex I inhibitor [54]

Complications of usage of IDH1 inhibitors

Ivosidenib is commonly associated with non-fatal, low-grade adverse effects such as nausea, vomiting, diarrhoea, and headaches [21-25]. These have been observed in patients involved in most of the clinical trials and have not caused cessation of therapy [21-25]. The following is a list of adverse effects that tend to be life-threatening and need to be dealt with at the earliest.

Cardiovascular Complications

In a study performed by Janus et al., 94,626 adverse effects reported during the management of AML with different drugs were analyzed, and a total of 675 pericardial adverse events, 479 tamponades, and 145 myocarditis events were observed [56]. Ivosidenib was responsible for 0.3% of pericardial side effects and was significantly safer when compared to cytarabine (0.9%, p=0.02). The incidence of tamponade was also significantly lower with ivosidenib when compared to cytarabine. This shows that ivosidenib is a safer drug, but it has to be noted that cardiac toxicity is possible and has to be watched out for in patients who are at risk [56]. QTc prolongation has also been reported in a few patients and is to be considered a serious complication [57]. Hence, concomitant use of ivosidenib with drugs that prolong the QT interval should be avoided. Ivosidenib is to be discontinued if QTc > 480 ms and is to be restarted only after QTc values return to less than or equal to 480 ms [57].

Dermatologic Complications

In an analysis by Parisi et al., a total of 66 patients who were treated with ivosidenib and 103 patients treated with enasidenib were studied for dermatologic adverse effects (DAEs). The majority of patients had relapsed or refractory AML. Over 20% on ivosidenib and over 40% on enasidenib experienced at least 1 DAE. The grade of DAE was not associated with leukaemia severity or mortality. The median cumulative dose at the first DAE for ivosidenib was 17,500 mg, and the median cumulative dose at the first dose interruption was 42,000 mg. The most common DAEs were inflammatory dermatoses, cutaneous vascular manifestations, cutaneous infections, and pruritis [58].

The most common inflammatory dermatoses were maculopapular rash, dermatitis, non-infectious neutrophilic dermatoses, leukaemia cutis, and exacerbations of pre-existing dermatoses. About 15% of them were high-grade. The mean onset of maculopapular rash following ivosidenib administration was 38 days (range: 18-94 days). Despite high-grade rashes, they rarely lead to the discontinuation of treatment [58]. Common vascular DAEs include oedema (the most common), hematomas, petechiae, and superficial thrombophlebitis. Among cutaneous infections, cellulitis was the most common, and this rarely caused treatment cessation. Other DAEs include hirsutism, hyperhidrosis, pallor, photosensitivity, and post-inflammatory hyperpigmentation [58]. Cutaneous manifestations of systemic differentiation syndrome (DS) were observed in four patients. Cases of IDH1 inhibitor-induced neutrophilic dermatosis have also been reported [59].

IDH-Induced Differentiation Syndrome

DS is a clinical syndrome first described in patients with acute promyelocytic leukaemia treated with all-trans-retinoic acid (ATRA) [60]. Similar to the cellular arrest of differentiation as observed in mIDH1/2 AML due to the accumulation of 2-HG and other factors, cells are arrested in primitive stages in APL too [15-17]. ATRA accelerates differentiation and leads to a sudden rise in WBC counts during the initial stages of treatment [60]. Excessive mature white blood cells can cause cytokine storms, infiltration into the lung parenchyma and other extravascular spaces, and inflammation leading to a systemic syndrome [60]. Symptoms commonly include dyspnea, fever, weight gain, unexplained hypotension, acute renal failure, and chest radiographs demonstrating pulmonary infiltrates or pleuropericardial effusion [60]. Patients with two or three of the above-mentioned signs/symptoms are classified as having moderate DS, and those with at least four are classified as having severe DS [61].

As described earlier in the review, ivosidenib also releases the differentiation block by reducing 2-HG production and hence can cause DS in vulnerable patients [15-17]. The incidence of DS in patients with relapsed, refractory AML treated with ivosidenib or enasidenib ranges between 11% and 14%, which makes it a very significant complication [22]. In a systematic review performed by Norsworthy et al., the incidence of DS due to ivosidenib was around 19% [62]. Baseline peripheral blood and bone marrow blast percentages greater than the median for both drugs (peripheral blasts ≥25%, bone marrow blasts ≥48%) have been shown to be associated with a higher risk of developing DS. Relapsed, refractory AML patients with a TET2 mutation co-existing with mIDH1/2 were also associated with a higher risk of developing DS [62]. The median time to the onset of DS was 20 days. The most common symptoms encountered were dyspnea and pulmonary infiltrates, or pleuropericardial effusion. This shows that DS can be a dangerous disease that has to be prevented. About 12% of the patients had repeat episodes of DS, which makes it important to closely monitor a patient with a past history of DS. The study has concluded that most patients on ivosidenib experience two episodes. DS also has a negative impact on the median overall survival (OS) and median duration of response (DOR). The median OS with versus without DS was 4.7 versus 10.0 months. There was a significant shortening of the median duration of IDH inhibitor response in patients with DS when compared to those without DS (3.2 months vs. 4.2 months) [62]. Systemic corticosteroids, especially dexamethasone, have been the drug of choice for the management of DS in the majority of cases, depending on the severity. Hydroxyurea and furosemide have also been used [62].

Infectious Complications

Ivosidenib has not been known to have gross immunosuppressive effects and hence rarely results in infections [63]. In a phase 1 study of ivosidenib monotherapy, febrile neutropenia was seen in 25.2% and pneumonia in 14.7%, but there was no association with the study drug [22]. In 179 patients treated with ivosidenib 500 mg, febrile neutropenia was noted in 28.5% of patients, and in a subgroup of 34 patients who were ineligible for standard chemotherapy and were treated with ivosidenib 500 mg, febrile neutropenia was seen in three patients and pneumonia in three patients [21]. Drugs being used for the management of infections are to be carefully considered while concomitantly using ivosidenib [63]. Most of the commonly used antimicrobial agents, such as anti-fungal azoles and fluoroquinolones, are CYP3A4 inhibitors and can cause QTc prolongation. As mentioned earlier, ivosidenib is metabolized by CYP3A4 and is also associated with QTc prolongation [57,63]. It was observed that the area under the curve (AUC) of ivosidenib increased by 169 when co-administered with itraconazole and 73% when co-administered with fluconazole [64]. Hence, care has to be taken while managing infections by monitoring the ECG of the patient to ensure healthy cardiac status [57]. In patients who need antifungal therapy, alternative treatments such as aerosolized liposomal amphotericin B should be considered in addition to systemic anti-fungal treatment [65,66]. Other anti-fungal agents that can be used include echinocandins or low-dose amphotericin B. Isavuconazole as an oral agent is also an option that can be used [67]. While prescribing ivosidenib alongside a strong CYP3A4 inhibitor, a dose reduction of ivosidenib from 500 mg/day to 250 mg/day is recommended to prevent complications [68].

Future prospects

A major issue troubling every physician treating AML is the rising incidence of ivosidenib resistance [26]. The mechanisms responsible for it have been described in detail earlier. Most of the future prospects revolve around combination therapies and making the drug safer and more efficacious. A deeper analysis and clinical trial into combining ivosidenib with conventional systemic chemotherapeutic regimens has to be conducted to understand the role mIDH1/2 plays in disease prognosis [24]. Combinations with other targeted inhibitors, such as FLT3 inhibitors (midostaurin), also need to be considered seriously in view of the rising incidence of AML with multiple genetic mutations [28].

## Conclusions

Ivosidenib has revolutionized the management of mIDH1 AML and has shown promising, prolonged, and durable responses. It changed the fate of patients with relapsed or refractory AML by creating a treatment avenue that was not available in the past. But there are significant hurdles that pose a challenge ahead, and a huge one is ivosidenib resistance. Although resistance mechanisms are being studied actively and a good number of them can be tackled, certain processes, such as excessive mitochondrial metabolism, are difficult to deal with effectively. Nonetheless, the improvement in OS that ivosidenib has shown has been remarkable and has once again proven that targeted and personalized patient therapy are the two growing avenues in the branches of haematology and oncology that have a great potential to improve patients’ survival rates.

## References

[1] Alabduladhem TO, Bordoni B (2023). Physiology, Krebs Cycle. https://pubmed.ncbi.nlm.nih.gov/32310492/.

[2] Ying W (2008). NAD+/NADH and NADP+/NADPH in cellular functions and cell death: regulation and biological consequences. Antioxid Redox Signal.

[3] Zarei M, Hue JJ, Hajihassani O (2022). Clinical development of IDH1 inhibitors for cancer therapy. Cancer Treat Rev.

[4] Dang L, Yen K, Attar EC (2016). IDH mutations in cancer and progress toward development of targeted therapeutics. Ann Oncol.

[5] Montalban-Bravo G, DiNardo CD (2018). The role of IDH mutations in acute myeloid leukemia. Future Oncol.

[6] Dang L, Jin S, Su SM (2010). IDH mutations in glioma and acute myeloid leukemia. Trends Mol Med.

[7] Urban DJ, Martinez NJ, Davis MI (2017). Assessing inhibitors of mutant isocitrate dehydrogenase using a suite of pre-clinical discovery assays. Sci Rep.

[8] Craddock CF, Houlton AE, Quek LS (2017). Outcome of azacitidine therapy in acute myeloid leukemia is not improved by concurrent vorinostat therapy but is predicted by a diagnostic molecular signature. Clin Can Res.

[9] DiNardo CD, Ravandi F, Agresta S (2015). Characteristics, clinical outcome, and prognostic significance of IDH mutations in AML. Am J Hematol.

[10] Tap WD, Villalobos VM, Cote GM (2020). Phase I study of the mutant IDH1 inhibitor ivosidenib: safety and clinical activity in patients with advanced chondrosarcoma. J Clin Oncol.

[11] Boscoe AN, Rolland C, Kelley RK (2019). Frequency and prognostic significance of isocitrate dehydrogenase 1 mutations in cholangiocarcinoma: a systematic literature review. J Gastrointest Oncol.

[12] Shibata T, Kokubu A, Miyamoto M, Sasajima Y, Yamazaki N (2011). Mutant IDH1 confers an in vivo growth in a melanoma cell line with BRAF mutation. Am J Pathol.

[13] Abou-Alfa GK, Macarulla T, Javle MM (2020). Ivosidenib in IDH1-mutant, chemotherapy-refractory cholangiocarcinoma (ClarIDHy): a multicentre, randomised, double-blind, placebo-controlled, phase 3 study. Lancet Oncol.

[14] Mellinghoff IK, Ellingson BM, Touat M (2020). Ivosidenib in isocitrate dehydrogenase 1-mutated advanced glioma. J Clin Oncol.

[15] Dang L, White DW, Gross S (2009). Cancer-associated IDH1 mutations produce 2-hydroxyglutarate. Nature.

[16] Jiang L, Shestov AA, Swain P (2016). Reductive carboxylation supports redox homeostasis during anchorage-independent growth. Nature.

[17] Galluzzi L, Kroemer G (2018). Potent immunosuppressive effects of the oncometabolite R-2-hydroxyglutarate. Oncoimmunology.

[18] Chen Y, Nagaraja NV, Fan B (2021). Preclinical drug metabolism, pharmacokinetic, and pharmacodynamic profiles of ivosidenib, an inhibitor of mutant isocitrate dehydrogenase 1 for treatment of isocitrate dehydrogenase 1-mutant malignancies. Drug Metab Dispos.

[19] (2023). FDA approves ivosidenib for advanced or metastatic cholangiocarcinoma. https://www.fda.gov/drugs/resources-information-approved-drugs/fda-approves-ivosidenib-advanced-or-metastatic-cholangiocarcinoma.

[20] Norsworthy KJ, Luo L, Hsu V (2019). FDA approval summary: Ivosidenib for relapsed or refractory acute myeloid leukemia with an isocitrate dehydrogenase-1 mutation. Clin Cancer Res.

[21] Roboz GJ, DiNardo CD, Stein EM (2020). Ivosidenib induces deep durable remissions in patients with newly diagnosed IDH1-mutant acute myeloid leukemia. Blood.

[22] DiNardo CD, Stein EM, de Botton S (2018). Durable remissions with ivosidenib in IDH1-mutated relapsed or refractory AML. N Engl J Med.

[23] Montesinos P, Recher C, Vives S (2022). Ivosidenib and azacitidine in IDH1-mutated acute myeloid leukemia. N Engl J Med.

[24] Stein EM, DiNardo CD, Fathi AT (2021). Ivosidenib or enasidenib combined with intensive chemotherapy in patients with newly diagnosed AML: a phase 1 study. Blood.

[25] Genthon A, Dragoi D, Memoli M (2022). Isocitrate dehydrogenase inhibitors as a bridge to allogeneic stem cell transplant in relapsed or refractory acute myeloid leukaemia. Br J Haematol.

[26] Yao K, Liu H, Yu S, Zhu H, Pan J (2022). Resistance to mutant IDH inhibitors in acute myeloid leukemia: Molecular mechanisms and therapeutic strategies. Cancer Lett.

[27] Choe S, Wang H, DiNardo CD (2020). Molecular mechanisms mediating relapse following ivosidenib monotherapy in IDH1-mutant relapsed or refractory AML. Blood Adv.

[28] Fathi AT, Perl AE, Levis M (2020). Concurrent FLT3 inhibitor and IDH inhibitor therapy in patients with acute myeloid leukemia (AML). Blood Suppl.

[29] Amatangelo MD, Quek L, Shih A (2017). Enasidenib induces acute myeloid leukemia cell differentiation to promote clinical response. Blood.

[30] Degirmenci U, Wang M, Hu J (2020). Targeting aberrant Ras/Raf/MEK/ERK signaling for cancer therapy. Cells.

[31] Najafi M, Ahmadi A, Mortezaee K (2019). Extracellular-signal-regulated kinase/mitogen-activated protein kinase signaling as a target for cancer therapy: an updated review. Cell Biol Int.

[32] DiNardo CD, Stein EM (2018). Soho state of the art update and next questions: IDH therapeutic targeting in AML. Clin Lymphoma Myeloma Leuk.

[33] Linos K, Tafe LJ (2018). Isocitrate dehydrogenase 1 mutations in melanoma frequently co-occur with NRAS mutations. Histopathology.

[34] Wang F, Morita K, DiNardo CD (2021). Leukemia stemness and co-occurring mutations drive resistance to IDH inhibitors in acute myeloid leukemia. Nat Commun.

[35] Brown AL, Hahn CN, Scott HS (2020). Secondary leukemia in patients with germline transcription factor mutations (RUNX1, GATA2, CEBPA). Blood.

[36] DiNardo CD, Cortes JE (2016). Mutations in AML: prognostic and therapeutic implications. Hematology Am Soc Hematol Educ Program.

[37] Caiado F, Maia-Silva D, Jardim C (2019). Lineage tracing of acute myeloid leukemia reveals the impact of hypomethylating agents on chemoresistance selection. Nat Commun.

[38] Wu J, Izpisua Belmonte JC (2016). Stem cells: a renaissance in human biology research. Cell.

[39] Figueroa ME, Abdel-Wahab O, Lu C (2010). Leukemic IDH1 and IDH2 mutations result in a hypermethylation phenotype, disrupt TET2 function, and impair hematopoietic differentiation. Cancer Cell.

[40] Gatto L, Franceschi E, Tosoni A, Di Nunno V, Maggio I, Lodi R, Brandes AA (2021). IDH inhibitors and beyond: the cornerstone of targeted glioma treatment. Mol Diagn Ther.

[41] Oltvai ZN, Harley SE, Koes D (2021). Assessing acquired resistance to IDH1 inhibitor therapy by full-exon IDH1 sequencing and structural modeling. Cold Spring Harb Mol Case Stud.

[42] Cho YS, Levell JR, Liu G (2017). Discovery and evaluation of clinical candidate IDH305, a brain penetrant mutant IDH1 inhibitor. ACS Med Chem Lett.

[43] Caravella JA, Lin J, Diebold RB (2020). Structure-based design and identification of FT-2102 (Olutasidenib), a potent mutant-selective IDH1 inhibitor. J Med Chem.

[44] Nathan B, Robin D, Serge B (2019). Identification and characterization of LY3410738, a novel covalent inhibitor of cancer-associated mutant Isocitrate Dehydrogenase 1 (IDH1). Cancer Res.

[45] Wang Z, Zhang Z, Li Y (2021). Preclinical efficacy against acute myeloid leukaemia of SH1573, a novel mutant IDH2 inhibitor approved for clinical trials in China. Acta Pharm Sin B.

[46] Harding JJ, Lowery MA, Shih AH (2018). Isoform switching as a mechanism of acquired resistance to mutant isocitrate dehydrogenase inhibition. Cancer Discov.

[47] Morita K, Wang F, Jahn K (2020). Clonal evolution of acute myeloid leukemia revealed by high-throughput single-cell genomics. Nat Commun.

[48] Vosberg S, Greif PA (2019). Clonal evolution of acute myeloid leukemia from diagnosis to relapse. Genes Chromosomes Cancer.

[49] Quek L, David MD, Kennedy A (2018). Clonal heterogeneity of acute myeloid leukemia treated with the IDH2 inhibitor enasidenib. Nat Med.

[50] Izquierdo-Garcia JL, Cai LM, Chaumeil MM (2014). Glioma cells with the IDH1 mutation modulate metabolic fractional flux through pyruvate carboxylase. PLoS One.

[51] Chan SM, Thomas D, Corces-Zimmerman MR (2015). Isocitrate dehydrogenase 1 and 2 mutations induce BCL-2 dependence in acute myeloid leukemia. Nat Med.

[52] Baccelli I, Gareau Y, Lehnertz B (2019). Mubritinib targets the electron transport chain complex I and reveals the landscape of OXPHOS dependency in acute myeloid leukemia. Cancer Cell.

[53] Grassian AR, Parker SJ, Davidson SM (2014). IDH1 mutations alter citric acid cycle metabolism and increase dependence on oxidative mitochondrial metabolism. Cancer Res.

[54] Stuani L, Sabatier M, Saland E (2021). Mitochondrial metabolism supports resistance to IDH mutant inhibitors in acute myeloid leukemia. J Exp Med.

[55] Liu AC, Cathelin S, Yang Y (2022). Targeting Stat5 signaling overcomes resistance to IDH inhibitors in acute myeloid leukemia through suppression of stemness. Cancer Res.

[56] Janus SE, Heisler AC, Al Jammal M (2022). Reported pericardial toxicities associated with acute myelogenous leukemia treatments: a pharmacovigilance analysis of the FDA adverse reporting database. Curr Probl Cardiol.

[57] Stemer G, Rowe JM, Ofran Y (2021). Efficacy and safety profile of ivosidenib in the management of patients with acute myeloid leukemia (AML): an update on the emerging evidence. Blood Lymphat Cancer.

[58] Parisi R, Cowen EA, Stoll JR (2022). Dermatologic adverse events associated with IDH inhibitors ivosidenib and enasidenib for the treatment of acute myeloid leukemia. Leuk Res.

[59] Dunn-Valadez S, Bathini S, Elston C (2022). IDH1 inhibitor-induced neutrophilic dermatosis in a patient with acute myeloid leukemia. Cancer Treat Res Commun.

[60] Frankel SR, Eardley A, Lauwers G, Weiss M, Warrell RP Jr (1992). The "retinoic acid syndrome" in acute promyelocytic leukemia. Ann Intern Med.

[61] Montesinos P, Bergua JM, Vellenga E (2009). Differentiation syndrome in patients with acute promyelocytic leukemia treated with all-trans retinoic acid and anthracycline chemotherapy: characteristics, outcome, and prognostic factors. Blood.

[62] Norsworthy KJ, Mulkey F, Scott EC (2020). Differentiation syndrome with ivosidenib and enasidenib treatment in patients with relapsed or refractory IDH-mutated AML: a U.S. Food and Drug Administration systematic analysis. Clin Cancer Res.

[63] Maschmeyer G, Bullinger L, Garcia-Vidal C (2022). Infectious complications of targeted drugs and biotherapies in acute leukemia. Clinical practice guidelines by the European Conference on Infections in Leukemia (ECIL), a joint venture of the European Group for Blood and Marrow Transplantation (EBMT), the European Organization for Research and Treatment of Cancer (EORTC), the International Immunocompromised Host Society (ICHS) and the European Leukemia Net (ELN). Leukemia.

[64] Stemler J, Jonge N, Skoetz N (2022). Antifungal prophylaxis in adult patients with acute myeloid leukaemia treated with novel targeted therapies: a systematic review and expert consensus recommendation from the European Hematology Association. Lancet Haematol.

[65] Maertens JA, Girmenia C, Brüggemann RJ (2018). European guidelines for primary antifungal prophylaxis in adult haematology patients: summary of the updated recommendations from the European Conference on Infections in Leukaemia. J Antimicrob Chemother.

[66] Safdar A, Rodriguez GH (2013). Aerosolized amphotericin B lipid complex as adjunctive treatment for fungal lung infection in patients with cancer-related immunosuppression and recipients of hematopoietic stem cell transplantation. Pharmacotherapy.

[67] Megías-Vericat JE, Solana-Altabella A, Ballesta-López O, Martínez-Cuadrón D, Montesinos P (2020). Drug-drug interactions of newly approved small molecule inhibitors for acute myeloid leukemia. Ann Hematol.

[68] Dai D, Yang H, Nabhan S (2019). Effect of itraconazole, food, and ethnic origin on the pharmacokinetics of ivosidenib in healthy subjects. Eur J Clin Pharmacol.

